# The Y-Encoded Gene *Zfy2* Acts to Remove Cells with Unpaired Chromosomes at the First Meiotic Metaphase in Male Mice

**DOI:** 10.1016/j.cub.2011.03.057

**Published:** 2011-05-10

**Authors:** Nadège Vernet, Shantha K. Mahadevaiah, Obah A. Ojarikre, Guy Longepied, Haydn M. Prosser, Allan Bradley, Michael J. Mitchell, Paul S. Burgoyne

**Affiliations:** 1 Division of Stem Cell Biology and Developmental Genetics, Medical Research Council National Institute for Medical Research, Mill Hill, London NW7 1AA, UK; 2 The Wellcome Trust Sanger Institute, Wellcome Trust Genome Campus, Hinxton, Cambridge CB10 1SA, UK; 3 Inserm UMRS 910, Faculté de Médecine Timone, Université de la Méditerranée, 27 Boulevard Jean Moulin, Marseille 13385, France

## Abstract

During male but not female mammalian meiosis, there is efficient apoptotic elimination of cells with unpaired (univalent) chromosomes at the first meiotic metaphase (MI) [1]. Apoptotic elimination of MI spermatocytes is seen in response to the univalent X chromosome of X*Sxr^a^*O male mice [2], in which the X chromosome carries *Sxr^a^* [3, 4], the Y-chromosome-derived sex-reversal factor that includes the testis determinant *Sry*. *Sxr^b^* is an *Sxr^a^-*derived variant in which a deletion has removed six Y short-arm genes and created a *Zfy2/Zfy1* fusion gene spanning the deletion breakpoint [4, 5]. X*Sxr^b^*O males have spermatogonial arrest that can be overcome by the re-addition of *Eif2s3y* from the deletion as a transgene; however, X*Sxr^b^*O*Eif2s3y* transgenic males do not show the expected elimination of MI spermatocytes in response to the univalent [6]. Here we show that these X*Sxr^b^*O*Eif2s3y* males have an impaired apoptotic response with completion of the first meiotic division, but there is no second meiotic division. We then show that *Zfy2* (but not the closely related *Zfy1*) is sufficient to reinstate the apoptotic response to the X univalent. These findings provide further insight into the basis for the much lower transmission of chromosomal errors originating at the first meiotic division in men than in women [7].

## Highlights

► An MI spindle assembly checkpoint response without MI arrest ► A Y chromosome gene triggers MI apoptosis ► *Zfy2* but not *Zfy1* restores MI apoptosis

## Results

**Figure 1:**
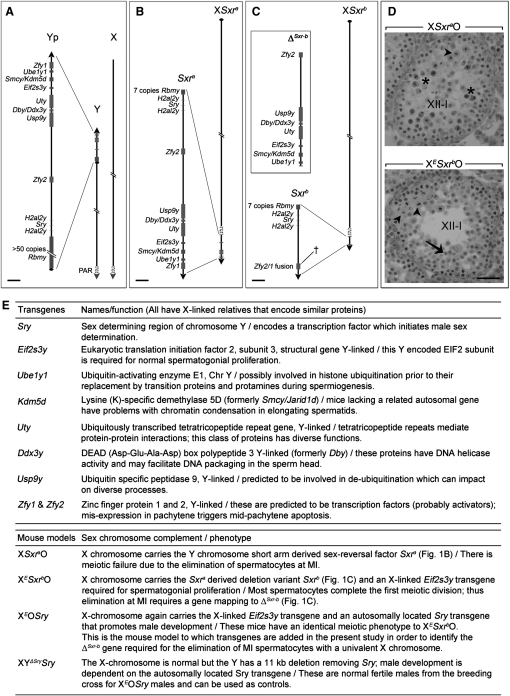
Information Pertaining to the Mouse Models (A) The mouse Y short arm (Yp; represented to scale in the magnified view) has seven single-copy genes, two duplicated genes, and one multi-copy gene. (Note that the mouse Y chromosome has a single distal pseudoautosomal region [PAR] on the long arm.) (B) The Y chromosome short-arm-derived *Sxr^a^* sex-reversal factor, here attached distal to the PAR on the X chromosome, includes most of the Yp genes. (C) The *Sxr^a^-*derived deletion variant *Sxr^b^* has a >900 kb deletion removing six single-copy genes (Δ*^Sxr-b^*) and creating a *Zfy2/1* fusion gene spanning the deletion breakpoint (†). (D) H & E-stained stage XII-I testis tubule section from 30-day-old X*Sxr^a^*O and X*^E^Sxr^b^*O mice. In X*Sxr^a^*O there are many arrested, darkly stained (dying; stars), MI spermatocytes. In X*^E^Sxr^b^*O most of the MI spermatocytes have already divided to form interphasic secondary spermatocytes (arrow), and those that remain are lightly stained (healthy; arrowheads). (E) Tables describing the transgenes and the mouse models analyzed in this study. The scale bar for magnified views in (A), (B), and (C) represents 150 Kb; it represents 40 μm in (D). See also Figure S1.

**Figure 2:**
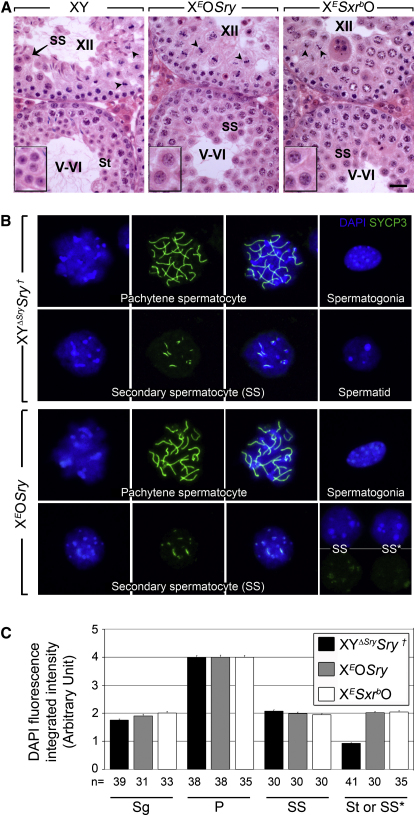
The Secondary-Spermatocyte Stage Is Reached in XO *Eif2s3y* Rescue Mice (A) H & E-stained stage XII and V–VI testis tubule sections from 30-day-old XY, X*^E^*O*Sry*, and X*^E^Sxr^b^*O mice. In XY males the two meiotic divisions occur within stage XII. Metaphase plates (arrowheads) and a pair of interphasic secondary spermatocytes (SS) are indicated. At stage V–VI, small, round haploid spermatids (St) are seen adjacent to the tubule lumen. In X*^E^*O*Sry* and X*^E^Sxr^b^*O males metaphase plates (arrowheads) are again present at stage XII; there are also some darkly stained cells or groups of cells that are indicative of cell death. Haploid spermatids are not found at stage V–VI; instead, the cells adjacent to the lumen are larger and have the morphology of interphasic secondary spermatocytes (SS). An example of spermatids or secondary spermatocytes is represented at a higher magnification in the left bottom part of each panel. The scale bar represents 20 μm. (B) Spread spermatogenic cells from 28- to 30-day-old XY^Δ*Sry*^*Sry* and X*^E^*O*Sry* males stained with DAPI (blue) and an antibody against SYCP3 (green). The pachytene cells serve as a positive control for SYCP3 staining. There is no SYCP3 staining in spermatogonia. Interphasic secondary spermatocytes with bar-shaped SYCP3 staining remaining at the majority of chromocenters are found in both genotypes. An SYCP3-negative round spermatid nucleus is shown for the control male (these were not found in X*^E^*O*Sry* males). Cells with the DAPI nuclear morphology of interphasic secondary spermatocytes but with very weak (SS) or absent (SS^∗^) SYCP3 staining (lower right panel) are an additional abundant cell type in X*^E^*O*Sry* males. (C) Nuclear DNA content quantitated by integrated intensity measurement of DAPI fluorescence on spread spermatogenic cells from 28- to 30-day-old X*^E^*O*Sry*, X*^E^Sxr^b^*O, and XY^Δ*Sry*^*Sry* males. The values are adjusted to give an arbitrary value of 4 for pachytene nuclei. In XY^Δ*Sry*^*Sry* males the spermatogonia (Sg), pachytene spermatocytes (P), secondary spermatocytes (SS), and spermatids (St) have the expected DNA content of 2C, 4C, 2C, and 1C, respectively. For X*^E^*O*Sry* and X*^E^Sxr^b^*O, “SS” includes cells with strong and weak SCP3 staining, and “SS^∗^” includes those with no SYCP3 staining; both have a 2C DNA content, and no cells with a 1C DNA content were identified. Two mice were analyzed per genotype, and the number of cells quantified for each group is indicated. ^†^XY^Δ*Sry*^*Sry* is a fertile control male genotype that is generated by the cross that produces X*^E^*O*Sry* males.

Schematic diagrams of the Y gene complement of the mouse Y short arm and of *Sxr^a^* and *Sxr^b^* along with salient details of the mouse models analyzed in this study are provided in Figure 1. Our first objective was to fully characterize the stage of meiotic arrest in X*Sxr^b^*O*Eif2s3y* transgenic males, as well as in XO*Sry,Eif2s3y* transgenic males that have the minimum Y gene complement compatible with progression to the first meiotic metaphase. For this we have used the X-linked *Eif2s3y* transgene of Mazeyrat et al. [6] (this transgene is hereafter denoted by X*^E^*); because it is on the X chromosome, this transgene is silenced during pachytene by ‘meiotic sex chromosome inactivation’ (MSCI [8]) thus mimicking the pachytene silencing of the endogenous *Eif2s3y*. In normal males the two meiotic divisions are completed during stage XII of the spermatogenic epithelium, although rare apoptotic MI spermatocytes may be retained into stage I. Histologically (Figure 2A) it appeared that in the X*^E^*O*Sry* and X*^E^Sxr^b^*O males (‘XO *Eif2s3y* rescue males’) a substantial number of spermatocytes in stage XII tubules are completing the first meiotic division to form interphasic secondary spermatocytes (in normal males this is a very transitory stage between the two meiotic divisions). Examination of subsequent tubule stages (e.g tubules at V-VI) revealed an accumulation of cells that had the morphology of interphasic secondary spermatocytes and the absence of cells with the nuclear morphology and size of round spermatids (insets in Figure 2A). It has previously been shown that MI and interphasic secondary spermatocytes retain the synaptonemal complex protein SYCP3 adjacent to the centromeres, SYCP3 then disappears soon after the formation of round spermatids. A combination of staining with SYCP3 and the nuclear stain DAPI thus enables interphasic secondary spermatocyte nuclei to be specifically identified [9]. SYCP3/DAPI staining was therefore carried out on spread spermatogenic cells, and interphasic secondary spermatocytes were clearly identified in the XO *Eif2s3y* rescue males (see Figure 2B for X*^E^*O*Sry*). However, not all the cells with the interphasic secondary spermatocyte nuclear morphology retained an SYCP3 signal; we suspect that these are secondary spermatocytes that have been retained beyond the time that SYCP3 staining would normally have disappeared. Quantification of the DAPI signal in these spread spermatogenic cells showed that all the presumptive interphasic secondary spermatocytes have the expected 2C DNA content and that no haploid spermatids (1C DNA content) are being produced (Figure 2C). These results show that in XO *Eif2s3y* rescue males a substantial number of spermatocytes are completing the first meiotic division and that there is then arrest at the interphase between the first and second meiotic divisions.

**Figure 3:**
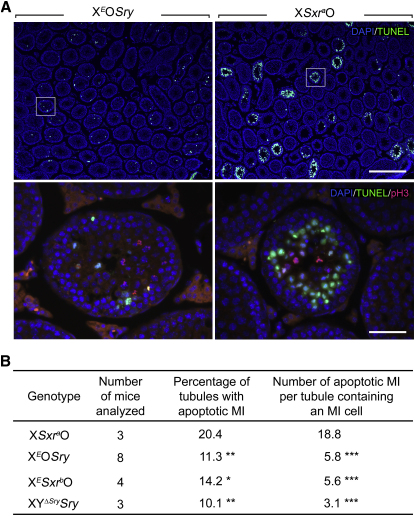
Markedly Reduced MI Apoptosis in XO *Eif2s3y* Rescue Mice (A) TUNEL-positive (green) first-meiotic metaphases (MIs) and healthy phosphor histone H3 (pH3, red) MIs were identified by their position away from the cell basal layers. DAPI (blue) was used as a nuclear stain. The upper panels show the paucity of apoptotic cells in 30 dpp X*^E^*O*Sry* compared to X*Sxr^a^*O mice. Below, at higher magnification, the boxed areas from the upper panels show the marked reduction of apoptotic MIs in stage XII tubules from X*^E^*O*Sry* mice. Healthy meiotic divisions are present in both genotypes. The scale bar represents 325 μm for the upper panels and 40 μm for the lower panels. (B) Quantitation of MI apoptosis was carried out on testis sections from 30-day-old X*Sxr^a^*O, X*^E^*O*Sry*, X*^E^Sxr^b^*O, and XY^Δ*Sry*^*Sry* mice. The latter three genotypes have significantly lower levels of apoptosis than X*Sxr^a^*O. ^∗^p ≤ 0.05, ^∗∗^p ≤ 0.01, and ^∗∗∗^p ≤ 0.001.

In X*Sxr^a^*O mice the spermatocytes are eliminated by apoptosis at MI [3, 10, 11]. Our confirmation that in X*^E^*O*Sry* and X*^E^Sxr^b^*O males substantial numbers of spermatocytes complete the first meiotic division implies that apoptosis is delayed or markedly reduced in these XO *Eif2s3y* rescue genotypes. To verify this we assessed MI apoptosis by TUNEL assay on X*^E^*O*Sry*, X*^E^Sxr^b^*O and X*Sxr^a^*O testis sections, carried out at 30 dpp in order to avoid the secondary germ cell loss that is known to occur as the XO *Eif2s3y* rescue males age [6]. This revealed that apoptotic elimination at MI is considerably reduced in the X*^E^*O*Sry* and X*^E^Sxr^b^*O males, there being a significant decrease in the percentage of tubules with MI spermatocyte apoptosis together with >3-fold decrease in the number of apoptotic MI spermatocytes in tubules with MI spermatocytes (Figures 3A and 3B). Nevertheless, some MI apoptosis still occurs in X*^E^*O*Sry* and X*^E^Sxr^b^*O (Figure 3B). Although the apoptotic response to X chromosome univalence is not totally abolished, there must be a Y gene mapping to the *Sxr^b^* deletion (Δ*^Sxr-b^*, inset in Figure 1C) that potentiates this response.

**Figure 4:**
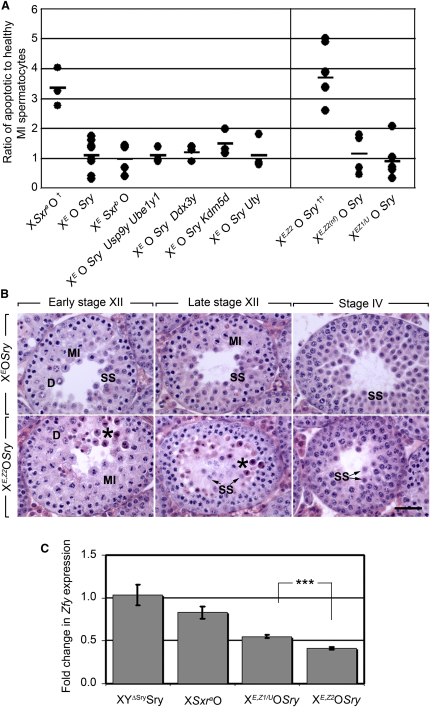
*Zfy2* Is the Y Gene that Maps to Δ*^Sxr-b^* and Reinstates the Apoptotic Elimination at MI (A) The ratio of apoptotic (TUNEL-positive) to healthy (pH3-positive) MI spermatocytes is plotted for each mouse, and the average ratio for each genotype is shown as a bar. Left panel: ratios for X*Sxr^a^*O, X*^E^*O*Sry*, and X*^E^Sxr^b^*O “controls” and for the X*^E^*O*Sry* mice with Δ*^Sxr-b^* genes added as transgenes. *Usp9y*, *Ube1y1*, *Ddx3y*, *Kdmd25*, and *Uty* did not reinstate the apoptotic elimination at MI. The right panel presents the results for the *Zfy* transgene addition: only the *Zfy2* transgene reinstates the apoptotic response. Importantly, a nonfunctional *Zfy2* transgene integrated at the same locus (X*^Z2(nf)^*) is unable to reinstate the MI apoptosis. ^†^The X*Sxr^a^*O males also carry the nonfunctional *Zfy2* transgene as a control for possible effects of the disruption of the *Hprt* locus. ^††^The apoptotic/healthy ratio for X*^E,Z2^*O*Sry* is significantly greater (p < 0.00001) than that for X*^E^*O*Sry* and not significantly different from that for X*Sxr^a^*O. (B) H & E-stained stage XII and stage IV testis tubule sections from 30-day-old X*^E^*O*Sry* and X*^E^*,^Z2^O*Sry* mice. Early and late stage XII testes were differentiated by the presence of diplotene (D) spermatocytes in the former and the lack of diplotene spermatocytes with the presence of secondary spermatocytes (SS) in the latter. In X*^E,Z2^*O*Sry* testes at stage XII there is a marked increase in MI spermatocytes that are heavily stained with eosin (starred), indicative of apoptosis; at stage IV there is a dramatic reduction in the number of secondary spermatocytes in comparison to X*^E^*O*Sry* testes. This reduction is due to the increased apoptotic elimination at MI in X*^E,Z2^*O*Sry* testes. The scale bar represents 40 μm. (C) Comparison by qRT-PCR of *Zfy* transcript levels in testes of different genotypes and between *Zfy1* and *Zfy2*; the primers used were common to both *Zfy* transcripts (a primer list is available in Table S1). Testes were collected when mice were 17.5 days old, when no overt differences in cell population were observed between genotypes; the germ-cell-specific transcript *Dazl* was used for normalization. XY*^ΔSry^Sry*, X*Sxr^a^*O, X*^EZ1/U^*O*Sry*, and X*^EZ2^*O*Sry* provide estimates of the levels deriving from *Zfy1*+*Zfy2* on the Y chromosome, *Zfy1*+*Zfy2* in *Sxr^a^*, *Zfy1* in the X*^Z1/U^* transgenic line, and *Zfy2* level in X*^Z2^* transgenic line. Relative transcript levels are expressed as the n-fold change ± SEM. The comparison of the two transgenic lines shows that *Zfy1* levels were significantly higher (^∗∗∗^p = 0.0001) than *Zfy2* levels. See also Figure S2.

Next we used transgene rescue with the X*^E^*O*Sry* model, to establish which gene(s) from Δ*^Sxr-b^* potentiates the apoptotic elimination at MI of cells with a univalent X chromosome. For this we initially used the *Ube1y1* and *Ddx3y* BAC transgenic lines used by Mazeyrat et al. [6] and newly generated BAC transgenic lines for *Kdm5d*, *Uty* and *Usp9y* [12]. These represent all the genes (in addition to *Eif2s3y*) that lie completely within the deletion. In a recent study the expression of all these transgenes was validated by RT-PCR and RNA FISH, the histology of XY testes with each transgene was shown to be grossly normal, and the males were fertile [12]. We produced 29-30 day-old X*^E^*O*Sry* mice with one or more of the transgenes for comparison with the X*^E^*O*Sry*, X*^E^Sxr^b^*O and X*Sxr^a^*O ‘controls’. For assessing the effect of the transgene additions on the apoptotic response at MI we used the TUNEL assay together with immunostaining for phospho histone H3 (pH3) in order to provide a measure of the ratio of apoptotic to healthy MI spermatocytes, as this provides a sensitive statistic for detecting changes in apoptotic rate (for details see methods). These ratios are >3-fold reduced in X*^E^*O*Sry* (1.1 ± 0.2) and X*^E^Sxr^b^*O (1.0 ± 0.3) as compared to X*Sxr^a^*O (3.4 ± 0.4). Classical histology showed that for all the transgene additions secondary spermatocytes are still abundant (data not shown); the combined TUNEL/pH3 assay comparisons showed there is no significant increase in the ratio of apoptotic to healthy MI spermatocytes (left panel Figure 4A). Thus the transgenic addition of *Usp9y*, *Ube1y1*, *Ddx3y*, *Kdm5d* or *Uty* to X*^E^*O*Sry* mice does not reinstate the apoptotic response.

In X*^E^Sxr^b^*O males *Zfy1* and *Zfy2* have been replaced by a transcribed *Zfy2/1* fusion gene spanning the Δ*^Sxr-b^* breakpoint [5]. This fusion gene has all the promoter elements of *Zfy2* but has the terminal exons 6-11 of *Zfy1* that encompass all but the first 20 amino acids of the open reading frame. It is therefore expected to express, with the pattern of expression of *Zfy2*, a protein identical to ZFY1 aside from the 16^th^ amino acid where a leucine is replaced by a phenylalanine (Figure S1). Prior to MI *Zfy1* and *Zfy2* have similar expression patterns [13, 14] so it is possible that the impaired MI apoptosis in the *Eif2s3y* rescue males is a consequence of a specific loss of *Zfy2* function; we therefore decided to introduce a *Zfy2* transgene into the X*^E^*O*Sry* mice.

Pronuclear injection of a *Zfy2* BAC leading to autosomal integration of the transgene proved to be incompatible with male fertility due to total apoptotic elimination of mid pachytene spermatocytes. This pachytene apoptosis is a consequence of the mis-expression of *Zfy2* during pachytene; *Zfy2* is silenced by MSCI when on the Y-chromosome [12]. To overcome this problem, we used cassette mediated exchange (CME) into the *Hprt* locus [15] to generate X-linked *Zfy2* transgenic lines in which the transgene would be subject to MSCI [12]. We generated two such lines and the carrier males were fertile; *Hprt* transcript analysis confirmed the expected CME disruption of the *Hprt* locus. However, only one of the lines expresses *Zfy2* (Figure S2), while in the second line the BAC proved to have a deletion removing the region encoding the *Zfy2* open reading frame (data not shown). We denote these functional and nonfunctional X-linked transgenes as X*^Z2^* and X*^Z2(nf)^* respectively; the nonfunctional *Zfy2* transgene provides a control for the loss of *Hprt* function associated with the CME insertion into the *Hprt* locus.

The histology of X*^E^*O*Sry* mice with the expressing *Zfy2* transgene (X*^E,Z2^*O*Sry*) showed a major reduction in the number of secondary spermatocytes, in conjunction with a marked increase in apoptotic MI spermatocytes (Figure 4B). We assessed apoptotic/healthy MI ratios using the TUNEL/pH3 assay and the ratio for X*^E,Z2^*O*Sry* mice (3.7 ± 0.3) was now equivalent to that obtained for X*Sxr^a^*O mice (3.4 ± 0.4), while the X*^Z2(nf)^* transgene had no effect (Figure 4A). Thus *Zfy2* reinstates the efficient apoptotic response to a univalent X chromosome. Subsequently a transgenic line with a single copy of a *Zfy1/Ube1y1* BAC integrated on the X (X*^Z1/U^*) by chance, was obtained following pronuclear injection [12]. We established by RT-PCR that this transgene expresses *Zfy1* (Figure S2), and by qRT-PCR using *Zfy2/1* common primers on testes from 17.5 day-old X*^E,Z1/U^*O*Sry* and X*^E,Z2^*O*Sry* males that *Zfy1* is present at a higher level than *Zfy2* (Figure 4C). Despite this higher expression, *Zfy1* does not reinstate the apoptotic response (Figure 4A). Thus there is a specific need for *Zfy2* for an efficient apoptotic response.

## Discussion

The efficient apoptotic elimination of MI spermatocytes with a univalent sex chromosome that fails to achieve bipolar attachment to the spindle (as in X*Sxr^a^*O males [2, 10, 11]) is widely assumed to be triggered by a spindle assembly checkpoint (SAC) monitoring the bipolar attachment of bivalents to the spindle [1]. This apoptotic response is male-specific as evidenced by the completion of both meiotic divisions in XO female mice [16]. Here we show that in mice the Y-encoded gene *Zfy2* (encoding a putative transcription factor) is required for this efficient apoptotic elimination at MI. It is interesting that *Zfy2* (together with *Zfy1*) was recently also shown to be responsible for the stage IV pachytene apoptosis associated with a failure to silence the Y chromosome during pachytene [12].

In the mouse the region of homology that mediates crossing over between the X and Y chromosomes (this region is called the pseudoautosomal region) only encompasses ∼700 kb of DNA [17], and a recent study has shown that efficient formation of a crossover in this region (on which the XY bivalent at MI depends) requires a late acting *Spo11* isoform that is dispensable for autosomal bivalent formation [18]. Therefore, might the *Zfy2*-dependent MI apoptotic response also be an adaptation allowing mice to cope with the risk of sex chromosome univalence? We have recently analyzed MI apoptosis in “Down syndrome mice” that carry a derivative of human chromosome 21 [19] and have found a >4-fold increase in apoptotic MI/healthy MI ratios compared to control ratios (XY = 0.30 ± 0.01; XY + h21 = 1.27 ± 0.1; NV and PSB, unpublished). Thus it is clear that the apoptotic response to univalence is not restricted to the sex chromosomes. It is well established that human trisomies (such as trisomy 21, Down syndrome) are predominantly due to maternal MI errors and that for most autosomal trisomies the incidence of these MI errors increases markedly with maternal age [7]. There is also accumulating evidence that the age-related increase is at least in part due to the progressive loss of the chiasmate links on which the bivalent association depends [20–22]; this can ultimately lead to separation into two univalents. However, it is also clear that pairs of homologous chromosomes that fail to establish a chiasmate link (non-exchange chromosomes), and thus will present as univalents at MI, are also associated with a preponderance of maternal MI errors but do not have the marked age dependence [23–25]. There is now substantial evidence for MI SAC activity in mammalian oocytes [26], so whatever the origin of the univalents it is expected that the majority will elicit an MI SAC response because they fail to achieve bipolar attachment to the spindle.

This still begs the question as to why the MI SAC response does not permanently arrest these cells at MI and thus prevent the transmission of aneuploidy. Significantly, in budding yeast engineered to have a pair of non-exchange chromosomes, there is only an approximately 1 hr SAC-mediated delay, after which the meiotic cycle resumes with the transmission of aneuploidy [27]. This parallels the lack of a meiotic block and the transmission of X monosomy by XO female mice, although no delay was detected with this single univalent [16]. During the first meiotic division in female mice depleted for the anaphase I promoting protein Cdc20, there is a high incidence of chromosome segregation errors involving one to a few bivalents, indicative of a failure to achieve bipolar attachment at MI; in this case there is an approximately 1–2 hr SAC-mediated delay, after which the meiotic divisions are completed and aneuploidy is transmitted [28]. Thus, although the MI SAC serves to allow time for all bivalents in chromosomally normal cells to achieve bipolar attachment to the spindle before proceeding to anaphase (avoiding the aneuploidy that results from premature anaphase entry), it does not permanently stall the meiotic division when faced with 1 or 2 obligate univalents or with a small number of bivalents that do not achieve bipolar attachment. Hence, the widely held view that the MI SAC should eliminate cells with 1 or a few univalents (which fail to achieve bipolar attachment) is unfounded. This view probably derives from observations of anaphase arrest after experimental interventions that result in *extensive* univalence, as well as from the belief that in males one or two univalents do cause a SAC-mediated arrest at MI. However, as we show here, in the absence of the apoptotic elimination, the first meiotic division is completed.

Why is it that males but not females have developed this efficient apoptotic response to MI univalence? From an evolutionary point of view it could be argued that there is not a strong selection for a mechanism to prevent monosomy or trisomy because the vast majority of monosomic and a substantial proportion of trisomic conceptions are lost very early in pregnancy [29], probably with little if any impact on subsequent fertility. In spermatogenesis cells pass through meiosis as synchronously developing cohorts of cells conjoined by intercellular bridges [30], so there may be a need to eliminate cells that begin to lag behind in their development. In this context it is significant that in mice lacking *Tex14*, which is essential for the formation of intercellular bridges between germ cells, females are fertile but males are sterile. The male sterility is due to apoptotic elimination of spermatocytes at the mid pachytene stage (spermatogonial apoptosis is at normal levels), and there is asynchrony in germ cell development within a tubule prior to this apoptosis [31, 32]. Mid-pachytene apoptosis is seen in the context of a number of meiotic mutants, many but not all of which also exhibit MSCI failure [33]. It has been suggested that this is a stage at which Sertoli cells “check” the spermatogenic epithelium for spermatocytes lagging behind the normal developmental schedule and trigger the apoptotic removal of the laggards [1, 34]. We suspect the MI apoptosis in response to the obligate univalent is triggered by a SAC-mediated delay.

In the mouse two Y-encoded copies of *Zfy* encode putative transcription factors expressed only in the testis. In man there is a single copy (*ZFY*) that is widely expressed, but there is a testis-specific splice variant (F. Decarpentrie and M.J.M., unpublished data). In mice, and presumably in humans, these Y-located genes are shut down in pachytene spermatocytes, and in mice we have found that transcription does not resume until after the first meiotic division. A challenge for the future will be to determine how *Zfy2* (but not *Zfy1*) is mechanistically linked to the apoptotic response and to determine whether there is a similar link with human *ZFY.*

## Supplemental Information

Document S1. Supplemental Experimental Procedures, Two Figures, and One Table

## References

[1] Burgoyne P.S., Mahadevaiah S.K., Turner J.M. (2009). The consequences of asynapsis for mammalian meiosis. Nat. Rev. Genet..

[2] Burgoyne P.S., Mahadevaiah S.K., Sutcliffe M.J., Palmer S.J. (1992). Fertility in mice requires X-Y pairing and a Y-chromosomal “spermiogenesis” gene mapping to the long arm. Cell.

[3] Cattanach B.M., Pollard C.E., Hawker S.G. (1971). Sex-reversed mice: XX and XO males. Cytogenetics.

[4] Mazeyrat S., Saut N., Sargent C.A., Grimmond S., Longepied G., Ehrmann I.E., Ellis P.S., Greenfield A., Affara N.A., Mitchell M.J. (1998). The mouse Y chromosome interval necessary for spermatogonial proliferation is gene dense with syntenic homology to the human AZFa region. Hum. Mol. Genet..

[5] Simpson E.M., Page D.C. (1991). An interstitial deletion in mouse Y chromosomal DNA created a transcribed Zfy fusion gene. Genomics.

[6] Mazeyrat S., Saut N., Grigoriev V., Mahadevaiah S.K., Ojarikre O.A., Rattigan A., Bishop C., Eicher E.M., Mitchell M.J., Burgoyne P.S. (2001). A Y-encoded subunit of the translation initiation factor Eif2 is essential for mouse spermatogenesis. Nat. Genet..

[7] Hassold T., Hunt P. (2001). To err (meiotically) is human: The genesis of human aneuploidy. Nat. Rev. Genet..

[8] Turner J.M. (2007). Meiotic sex chromosome inactivation. Development.

[9] Kudo N.R., Anger M., Peters A.H., Stemmann O., Theussl H.C., Helmhart W., Kudo H., Heyting C., Nasmyth K. (2009). Role of cleavage by separase of the Rec8 kleisin subunit of cohesin during mammalian meiosis I. J. Cell Sci..

[10] Kot M.C., Handel M.A. (1990). Spermatogenesis in XO, Sxr mice: Role of the Y chromosome. J. Exp. Zool..

[11] Sutcliffe M.J., Darling S.M., Burgoyne P.S. (1991). Spermatogenesis in XY, XYSxra and XOSxra mice: A quantitative analysis of spermatogenesis throughout puberty. Mol. Reprod. Dev..

[12] Royo H., Polikiewicz G., Mahadevaiah S.K., Prosser H., Mitchell M., Bradley A., de Rooij D.G., Burgoyne P.S., Turner J.M. (2010). Evidence that meiotic sex chromosome inactivation is essential for male fertility. Curr. Biol..

[13] Hansen M.A., Nielsen J.E., Tanaka M., Almstrup K., Skakkebaek N.E., Leffers H. (2006). Identification and expression profiling of 10 novel spermatid expressed CYPT genes. Mol. Reprod. Dev..

[14] Zambrowicz B.P., Findley S.D., Simpson E.M., Page D.C., Palmiter R.D. (1994). Characterization of the murine Zfy1 and Zfy2 promoters. Genomics.

[15] Prosser H.M., Rzadzinska A.K., Steel K.P., Bradley A. (2008). Mosaic complementation demonstrates a regulatory role for myosin VIIa in actin dynamics of stereocilia. Mol. Cell. Biol..

[16] LeMaire-Adkins R., Radke K., Hunt P.A. (1997). Lack of checkpoint control at the metaphase/anaphase transition: A mechanism of meiotic nondisjunction in mammalian females. J. Cell Biol..

[17] Perry J., Palmer S., Gabriel A., Ashworth A. (2001). A short pseudoautosomal region in laboratory mice. Genome Res..

[18] Kauppi L., Barchi M., Baudat F., Romanienko P.J., Keeney S., Jasin M. (2011). Distinct properties of the XY pseudoautosomal region crucial for male meiosis. Science.

[19] O'Doherty A., Ruf S., Mulligan C., Hildreth V., Errington M.L., Cooke S., Sesay A., Modino S., Vanes L., Hernandez D. (2005). An aneuploid mouse strain carrying human chromosome 21 with Down syndrome phenotypes. Science.

[20] Hodges C.A., Revenkova E., Jessberger R., Hassold T.J., Hunt P.A. (2005). SMC1beta-deficient female mice provide evidence that cohesins are a missing link in age-related nondisjunction. Nat. Genet..

[21] Lister L.M., Kouznetsova A., Hyslop L.A., Kalleas D., Pace S.L., Barel J.C., Nathan A., Floros V., Adelfalk C., Watanabe Y. (2010). Age-related meiotic segregation errors in mammalian oocytes are preceded by depletion of cohesin and Sgo2. Curr. Biol..

[22] Revenkova E., Eijpe M., Heyting C., Hodges C.A., Hunt P.A., Liebe B., Scherthan H., Jessberger R. (2004). Cohesin SMC1 beta is required for meiotic chromosome dynamics, sister chromatid cohesion and DNA recombination. Nat. Cell Biol..

[23] Hall H.E., Surti U., Hoffner L., Shirley S., Feingold E., Hassold T. (2007). The origin of trisomy 22: Evidence for acrocentric chromosome-specific patterns of nondisjunction. Am. J. Med. Genet. A..

[24] Oliver T.R., Feingold E., Yu K., Cheung V., Tinker S., Yadav-Shah M., Masse N., Sherman S.L. (2008). New insights into human nondisjunction of chromosome 21 in oocytes. PLoS Genet..

[25] Thomas N.S., Ennis S., Sharp A.J., Durkie M., Hassold T.J., Collins A.R., Jacobs P.A. (2001). Maternal sex chromosome non-disjunction: Evidence for X chromosome-specific risk factors. Hum. Mol. Genet..

[26] Yin S., Sun X.F., Schatten H., Sun Q.Y. (2008). Molecular insights into mechanisms regulating faithful chromosome separation in female meiosis. Cell Cycle.

[27] Cheslock P.S., Kemp B.J., Boumil R.M., Dawson D.S. (2005). The roles of MAD1, MAD2 and MAD3 in meiotic progression and the segregation of nonexchange chromosomes. Nat. Genet..

[28] Jin F., Hamada M., Malureanu L., Jeganathan K.B., Zhou W., Morbeck D.E., van Deursen J.M. (2010). Cdc20 is critical for meiosis I and fertility of female mice. PLoS Genet..

[29] Burgoyne P.S., Holland K., Stephens R. (1991). Incidence of numerical chromosome anomalies in human pregnancy estimation from induced and spontaneous abortion data. Hum. Reprod..

[30] Dym M., Fawcett D.W. (1971). Further observations on the numbers of spermatogonia, spermatocytes, and spermatids connected by intercellular bridges in the mammalian testis. Biol. Reprod..

[31] Greenbaum M.P., Yan W., Wu M.H., Lin Y.N., Agno J.E., Sharma M., Braun R.E., Rajkovic A., Matzuk M.M. (2006). TEX14 is essential for intercellular bridges and fertility in male mice. Proc. Natl. Acad. Sci. USA.

[32] Rezende C.A., Meistrich M.L., Matzuk M.M., Drumond A.L.A., Shetty G., Weng C.C., Chiarni-Garcia H. (2009). Asinchronism of spermatogenesis after intercellular bridges loss among germ cells in Tex mutant mice testis. Acta Microscopica.

[33] Mahadevaiah S.K., Bourc'his D., de Rooij D.G., Bestor T.H., Turner J.M., Burgoyne P.S. (2008). Extensive meiotic asynapsis in mice antagonises meiotic silencing of unsynapsed chromatin and consequently disrupts meiotic sex chromosome inactivation. J. Cell Biol..

[34] Barchi M., Mahadevaiah S., Di Giacomo M., Baudat F., de Rooij D.G., Burgoyne P.S., Jasin M., Keeney S. (2005). Surveillance of different recombination defects in mouse spermatocytes yields distinct responses despite elimination at an identical developmental stage. Mol. Cell. Biol..

