# Supplementary material for: The Y-Encoded Gene Zfy2 Acts to Remove Cells with Unpaired Chromosomes at the First Meiotic Metaphase in Male Mice
Source: Curr Biol. 2011 May 10;21(9):787–93. doi: 10.1016/j.cub.2011.03.057 (PMC3176893; doi:10.1016/j.cub.2011.03.057)
Supplement: Document S1. Supplemental Experimental Procedures, Two Figures, and One Table [file mmc1.pdf]

Current Biology Volume *21*

Supplemental Information

The Y-Encoded Gene *Zfy2* Acts to Remove Cells with Unpaired Chromosomes at the First Meiotic Metaphase in Male Mice

Nadège Vernet, Shantha K. Mahadevaiah, Obah A. Ojarikre, Guy Longepied, Haydn M. Prosser, Allan Bradley, Michael J. Mitchell, and Paul S. Burgoyne

```
Zfy1           MDEDEIELTP EEEKSFFDGI|GADAVHMDSD QIVVEVQETV FLANSDVTVH NFVPDNPGSV  60
Zfy2           MDEDEIELTP EEEKSLFDGI|GADAVHMDSD QISVEVQETV FLSNSDVTVH NFVPDDPDSV
Zfy2/1 Fusion  MDEDEIELTP EEEKSLFDGI|GADAVHMDSD QIVVEVQETV FLANSDVTVH NFVPDNPGSV

Zfy1           IIQDVIENVL IEDVHCSHIL EETDISDNVI IPEQVLNLGT AEEVSLAQFL IPDILTSGIT 120
Zfy2           IIQDVIENVL IEDVHCSHIL EETDISDNVI IPEQVLDLDT AEEVSLAQFL IPDILTSSIT
Zfy2/1 Fusion  IIQDVIENVL IEDVHCSHIL EETDISDNVI IPEQVLNLGT AEEVSLAQFL IPDILTSGIT

Zfy1           STSLTMPEHV LMSEAIHVSD VGHFEQVIHD SLVETEVITD PITADTSDIL VADCVSEAVL 180
Zfy2           STSLTMPEHV LMSEAIHVSN VGHFEQVIHD SLVEREIITD PLTADISDIL VADWASEAVL
Zfy2/1 Fusion  STSLTMPEHV LMSEAIHVSD VGHFEQVIHD SLVETEVITD PITADTSDIL VADCVSEAVL

Zfy1           DSSGMPLEQQ DNDKINCEDY LMMSLDEPSK ADLEGSSEVT MNAESGTDSS KLDEASPEVI 240
Zfy2           DSSGMPLEQQ DDARINCEDY LMMSLDEPSK TDHEGSSEVT MNAESETDSS KLDEASPEVI
Zfy2/1 Fusion  DSSGMPLEQQ DNDKINCEDY LMMSLDEPSK ADLEGSSEVT MNAESGTDSS KLDEASPEVI

Zfy1           KVCILKADSE VDELGETIHA VESETKNGNE AEVTDQSTSI RVPRVNIYMS ASDSQKEEED 300
Zfy2           KVCILKADSE VDDVGETIQA VESETDNGNE AEVTDQRTSI HVPRVNIYML ASDSQKEEED
Zfy2/1 Fusion  KVCILKADSE VDELGETIHA VESETKNGNE AEVTDQSTSI RVPRVNIYMS ASDSQKEEED

Zfy1           TEVIVGDEDA GGTAADTPEH EQQMDVSEIK AAFLPIAWTA AYDNNSDEIE DQNVTASALL 360
Zfy2           TKVIVGDEDA GGTAADTPEH EQQMDVSEIK AAFLPIAWTA AYDNNSDEIE VQNATASAML
Zfy2/1 Fusion  TEVIVGDEDA GGTAADTPEH EQQMDVSEIK AAFLPIAWTA AYDNNSDEIE DQNVTASALL

Zfy1           NQDESGGLDR VPKQKSKKKK RPESKQYQSA IFVAPDGQTL RVYPCMFCGK KFKTKRFLKR 420
Zfy2           HHDESGGLDR VPKQKSKKKK RPESKQYQSA IFVAPDGQTL RVYPCMFCGK KFKTKRFLKR
Zfy2/1 Fusion  NQDESGGLDR VPKQKSKKKK RPESKQYQSA IFVAPDGQTL RVYPCMFCGK KFKTKRFLKR

Zfy1           HTKNHPEYLA NKKYHCTECD YSTNKKISLH NHMESHKLTI KTEKTTECDD CRKNLSHAGT 480
Zfy2           HIKNHPEYLA NKKYHCTECD YSTNKKISLH NHMESHKLTI KTEKTTECDD CRKNLSHAG-
Zfy2/1 Fusion  HTKNHPEYLA NKKYHCTECD YSTNKKISLH NHMESHKLTI KTEKTTECDD CRKNLSHAGT

Zfy1           LCTHKTMHTE KGVNKTCKCK FCDYETAEQT LLNHHLLVVH RKKFPHICGE CGKGFRHPSA 540
Zfy2           -----TMHTE KGVNKTCKCK FCDYETAEQT LLNHHLLVVH RKKFPHICGE CGKGFRHPSA
Zfy2/1 Fusion  LCTHKTMHTE KGVNKTCKCK FCDYETAEQT LLNHHLLVVH RKKFPHICGE CGKGFRHPSA

Zfy1           LKKHIRVHTG EKPYECQYCE YKSADSSNLK THIKSKHSKE IPLKCGICLL TFSDNKEAQQ 600
Zfy2           LKKHIRVHTG EKPYECQYCE YKSADSSNLK THIKSKHSKE IPLKCGICLL TFSDTKEAQQ
Zfy2/1 Fusion  LKKHIRVHTG EKPYECQYCE YKSADSSNLK THIKSKHSKE IPLKCGICLL TFSDNKEAQQ

Zfy1           HAVLHQESRT HQCSHCNHKS SNSSDLKRHI ISVHTKAYPH KCDMCSKGFH RPSELKKHVA 660
Zfy2           HAVLHQESRT HQCSHCNHKS SNSSDLKRHI ISVHTKAYPH KCDMCSKGFH RPSELKKHVA
Zfy2/1 Fusion  HAVLHQESRT HQCSHCNHKS SNSSDLKRHI ISVHTKAYPH KCDMCSKGFH RPSELKKHVA

Zfy1           THKSKKMHQC RHCDFNSPDP FLLSHHILSA HTKNVPFKCK RCKKEFQQQC ELQTHMKTHS 720
Zfy2           THKSKKMHQC RHCDFKSPDP FLLSHHILSA HTKNVPFKCK RCKKEFQQQC ELQTHMKTHS
Zfy2/1 Fusion  THKSKKMHQC RHCDFNSPDP FLLSHHILSA HTKNVPFKCK RCKKEFQQQC ELQTHMKTHS

Zfy1           SRKVYQCEYC EYSTKDASGF KRHVISIHTK DYPHSCDFCK KGFRRPSEKN QHIMRHHK-V 780
Zfy2           SRKVYQCEYC EYSTKDASGF KRHVISIHTK DYPHRCDFCK KGFRRPSEKN QHIMRHHKEV
Zfy2/1 Fusion  SRKVYQCEYC EYSTKDASGF KRHVISIHTK DYPHSCDFCK KGFRRPSEKN QHIMRHHK-V

Zfy1           GLP
Zfy2           GLA
Zfy2/1 Fusion  GLP
```

Figure S1. Protein sequence alignment for *Zfy1* and *Zfy2* together with the *Zfy2/1* fusion gene that is present in Tp(Y)1Ct^{Sxr-b} carrier mice such as X*Sxr*^{b}O (Related to Figure 1C)
The predicted *Zfy1* and *Zfy2* encoded proteins share 94.1% identity. The amino acid differences between *Zfy1* and *Zfy2* protein sequence are colored blue and the 13 zinc finger are highlighted in grey. The presence of these zinc fingers supports the idea that the protein is a transcription factor. The break point of the fusion gene *Zfy2/1* (in the intron 5) is represented by a vertical bar. The single amino acid difference between the predicted proteins for *Zfy1* and *Zfy2/1* is colored red.

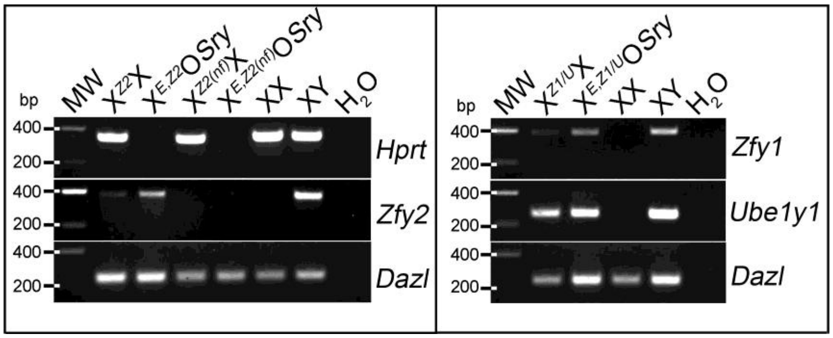

Figure S2. RT-PCR analysis for 30 day-old testes and 4-8 week-old ovaries from *Zfy2* and *Zfy1/Ube1y1* transgenic mice (Related to Figure 4A and Table S1)

The germ cell-specific transcript *Dazl* has been used as an amplification control. The left panel shows that for the two *Zfy2* lines (X^{Z2} and $X^{Z2(nf)}$), where the transgene has been integrated in the HPRT locus by cassette mediated exchange, we obtained the expected disruption of *Hprt* expression in $X^{E,Z2}$O*Sry* and $X^{E,Z2(nf)}$O*Sry* testes. No *Zfy2* transcript is detected in $X^{E,Z2(nf)}$O*Sry* testis due to a deletion within *Zfy2* that renders the gene nonfunctional. In ovaries with the functional *Zfy2* transgene, *Hprt* is transcribed from the normal X-chromosome and *Zfy2* is faintly expressed. The right panel shows the expression of *Zfy1* and *Ube1y1* in mice transgenic for a BAC containing *Zfy1* and *Ube1y1*, which integrated on the X-chromosome by chance ($X^{Z1/U}$). *Zfy1* and *Ube1y1* are expressed in $X^{EZ1/U}$O*Sry* testes and fainter in $X^{Z1/U}$X ovaries.

Gene	Product size	Primer sequences	Reference
Ube1y1	266 bp	UBE1Y1-FP1 CGACAGCAACTTTCACATGG<br>UBE1Y1-R GAGCCAGAGGTGCAGAAAAG	[1]
Zfy1	386 bp	ZFY1F GCCAGTGCTCTCTTAAACCAA<br>ZFY1R TGAGTACACAAAGTCCCAGCA	[1]
Zfy2	386 bp	ZFY2F GCCAGTGCTATGTTACACCAT<br>ZFY2R TCTGTATGCATTGTCCCAGCA	[1]
Zfy *	241 bp	ZFYCOMF1 TGGATGAAGCATCTCCAGAA<br>ZFYCOMR1 CCACCAGCATCTTCATCTCC	[1]
	173 bp	ZFY COM F3 GGCAATGAAGCTGAAGTCAC<br>ZFY COM R3 TCCATCTGTTGCTCATGCTC	[1]
Hprt	352 bp	HPRT-1A CCTGCTGGATTACATTAAAGCACTG<br>HPRT-1B GTCAAGGGCATATCCAACAACAAAC	[1]
Dazl	234 bp	DAZL-F CCTCCAACCATGATGAATCC<br>DAZL-R TGAACATTCATTGGGCAAAA	Current paper

* These *Zfy* primers amplify both *Zfy1* and *Zfy2*.

Table S1. List of primers used (Related to Figures 4C and S2)

Supplemental Experimental Procedures

Origin of mouse lines carrying genes mapping to the *Sxr^b* deletion (see text Figure 1)

All transgenic lines except the *Zfy2* lines were produced by microinjection of purified BAC DNA into fertilized eggs from CBA/Ca x C57Bl/10 F1s as previously described [1]. Four *Eif2s3y* transgenic lines were used in our original 2001 study that first generated XO *Eif2s3y* rescue mice [2]; one of these [Tg(*Eif2s3y*)6Bu] is X-linked, and we chose to use this for the present study because it is silenced during pachytene like the endogenous *Eif2s3y*. The transgene is present in ~10 copies resulting in a 10-fold increase in *Eif2s3y* transcript levels (our unpublished data). Nevertheless, XY male carriers are fully fertile with grossly normal testis histology. The *Ube1y1*, *Kdm5d* (formerly *Smcy*/*Jarid1d*), *Uty*, *Ddx3y* (formerly *Dby*), and *Usp9y* transgenes we used are autosomally located and have been fully characterized by Royo et al. [1]. For *Zfy1* we used a transgenic line with a *Zfy1+Ube1y1* BAC randomly inserted on the X-chromosome [1]. For the *Zfy2* and *Zfy2(nf)* lines, X-chromosome directed transgenesis was performed by cassette mediated exchange (CME) as previously described [1, 3]. Two transgenic mice lines were created one of which turned out to have a deletion in the BAC removing the region encoding the *Zfy2* open reading frame. We utilized this line with a non-functional *Zfy2(nf)* as a control for the loss of *Hprt* function associated with the CME insertion into the *Hprt* locus.

To produce the mice analyzed in the present study, we mated $X^{Paf}O$ females (carrying the X-linked *Patchy-fur* mutation as a coat marker) [4] to: (1) '$X^{E}Y$*Sxr^b*' males that have the X-linked *Eif2s3y* transgene [12] and a Y-chromosome that has the Tp(Y)1Ct$^{Sxr\text{-}b}$ sex-reversal factor [5] attached distal to its pseudoautosomal region; this cross produces X^{E}*Sxr^b*O males with a normal coat. (2) '$X^{E}Y^{\Delta Sry}$*Sry*' males that have the X carrying the *Eif2s3y* transgene

[2], a Y-chromosome with an 11 kb deletion removing *Sry* (*dl1Rlb*) [6-7], and an autosomally located *Sry* transgene [Tg(Sry)2Ei] [8]; this cross produces $X^{E}O$*Sry* males with a normal coat. (3) '$X^{E}Y^{\Delta Sry}$*Sry*' males that also carry one or more of the other transgenes from the Sxr^{b} deletion ($\Delta^{Sxr\text{-}b}$); these crosses generate the $X^{E}O$*Sry* males with additional $\Delta^{Sxr\text{-}b}$ genes. (4) '$X^{Z2(nf)}YSxr^{a}$' that have the nonfunctional X-linked *Zfy2* transgene inserted in the *Hprt* locus and a Y-chromosome that has the $Tp(Y)1Ct^{Sxr\text{-}a}$ sex-reversal factor [9] attached distal to its pseudoautosomal region; this cross produces $X^{Z2(nf)}Sxr^{a}O$ males with a normal coat. The mice were produced on the random bred albino MF1 stock background (NIMR colony); and in some cases MF1 XY males were used as controls. All animal procedures were in accordance with the United Kingdom Animal Scientific Procedures Act 1986 and were subject to local ethical review.

Histological analysis, immuno-fluorescence and TUNEL assays

For standard histological analysis testes were fixed in Bouin, embedded in paraffin, sectioned at 5 µm on glass slides that were then stained with Hematoxylin and Eosin (H & E). At least 4 males per genotype were analyzed.

For immuno-fluorescence and detection of apoptotic cells, testis samples were fixed in 4% PFA overnight at 4°C and embedded in paraffin. 5 µm sections on glass slides were de-waxed, washed in PBS, soaked in PBST-BSA for 1 hour, and then incubated overnight at 37°C with rabbit polyclonal anti-phospho-histone H3 (1:300; Upstate) diluted in PBST-BSA. Slides were then washed in PBST, incubated at 37°C for 1h with chicken anti-rabbit Alexa 594 (1:500; Molecular Probes) diluted in PBS, and then washed in PBST.

Apoptotic cells were detected using the terminal deoxynucleotidyl transferase dUTP nick end-labeling (TUNEL) assay (In Situ Cell Death Detection Kit®, fluorescein; Roche). Nuclei were counterstained with Vectashield containing 4',6-diamidino-2-phenylindole (DAPI, Vector). For quantitating the differences in apoptotic responses between the genotypes, it is important to realize that no method gives a measure of the proportion of cells that become apoptotic. This is because all MI's begin as healthy (leading to an underestimate of the proportion that become apoptotic), but also because those that become apoptotic are retained well beyond the time that the MIs that remain healthy have completed both divisions (resulting in a gross over estimate of the number of cells that are proceeding through MI [10]). Thus any method only gives a measure of the relative increase in the number of apoptotic cells. For the initial quantitation of apoptosis in $X^{E}O$*Sry* and X^{E}*Sxr*^{b}O males an entire median cross section of each testis was scanned to identify tubule cross sections with apoptotic (TUNEL positive) or healthy (pH3 positive) metaphases away from the basal layers of the tubules (in order to exclude spermatogonial metaphases) and the number of such metaphases in each tubule cross section was recorded; 170-250 tubule cross sections per mouse and at least 3 mice were analyzed per genotype. In order to analyze the effects of $\Delta^{Sxr\text{-}b}$ transgene addition to $X^{E}O$*Sry*, we calculated the ratio of apoptotic (TUNEL positive) to healthy (phospho histone H3 positive) metaphases at MI, to provide a sensitive statistic for detecting changes in apoptotic rate. ANOVA analysis (General linear models, NCSS, Kaysville, UT, USA) was carried out for the percentage of tubules with 1 or more apoptotic MI's (after angular transformation of percentages), the number of apoptotic MIs per tubule, and the apoptotic/healthy ratios.

Staging of the seminiferous epithelium

The cycle of the seminiferous epithelium is divided into 12 stages, each defined by a specific association of germ cells [11]. These epithelial stages are precisely delineated on H & E stained histological sections from Bouin-fixed testes, but this method has limitations when the round or elongating spermatids are missing. In these instances, we identified the tubules stage according to the layers of spermatogonia and primary spermatocytes as previously described [12].

Testis cell spreads and DNA content

Surface spread cells were prepared as previously described [13], with slight modifications. Briefly, frozen testicular tissue was defrosted and macerated in RPMI 1640 solution (Invitrogen Corporation, Gibco) to produce a thin cell suspension. One drop of cell suspension was mixed with one drop of 0.05% Triton X-100 solution on a pre-boiled microscope slide and allowed to stand for 10 min. Eight drops of 2% formaldehyde solution (TAAB) containing 0.02% SDS pH 8.4 were added for 30 min, after which the slides were dipped briefly in distilled water and air dried. After rehydration in PBS the slides were soaked in PBST-BSA for 1 hour and incubated overnight at 37°C with rabbit polyclonal anti-SYCP3 (1:100; Abcam) diluted in PBST-BSA. Slides were washed in PBST, incubated with chicken anti-rabbit Alexa 488 (1:500; Molecular Probes) diluted in PBS for 1 h at 37°C and washed in PBST. DNA content of testicular cells was analyzed by fluorescence intensity measurement on a Leica microscope after staining with DAPI diluted in the mounting medium (Vectashield with DAPI; Vector). Two mice per genotype have been used and images of over 30 cells per cell type (spermatogonia, spermatocytes, secondary spermatocytes and spermatids) have been captured and analyzed using ImageJ 1.41o. The

integrated intensity was corrected by the background intensity for each measurement. The different cell types were defined based on nuclear size, DAPI morphology, and the SYCP3 staining pattern.

RNA extraction, reverse transcription and quantitative PCR

Total RNA was extracted from frozen testis with Trizol (Invitrogen) and DNaseI-treated (Invitrogen) using standard protocols. Reverse transcription of polyadenylated RNA was performed with Superscript Reverse Transcriptase II, according to the manufacturer's protocols (Invitrogen). For regular PCR, 35 cycles were performed with a hybridization temperature of 60°C. Primer sequences are available in Table S1. For quantification of relative *Zfy* transcript levels in the transgenic lines, real-time PCR was performed as described before [14]. Two sets of common primers detecting both *Zfy1* and *Zfy2* were used. Samples from three 17.5 day-old mice for each genotype have been analyzed. All reactions were carried out in triplicate per assay and *Dazl* was included on every plate as a loading control. The difference in PCR cycles with respect to *Dazl* (ΔCt) for a given experimental sample was subtracted from the mean ΔCt of the reference samples ($XY^{\Delta Sry}$*Sry*) (ΔΔCt). The fold change in expression is calculated as the mean of the power 2(ΔΔCt) for each genotype.

Supplemental References

1. Royo, H., Polikiewicz, G., Mahadevaiah, S.K., Prosser, H., Mitchell, M., Bradley, A., de Rooij, D.G., Burgoyne, P.S., and Turner, J.M. (2010). Evidence that meiotic sex chromosome inactivation is essential for male fertility. Curr. Biol. *20*, 2117-2123.
2. Mazeyrat, S., Saut, N., Grigoriev, V., Mahadevaiah, S.K., Ojarikre, O.A., Rattigan, A., Bishop, C., Eicher, E.M., Mitchell, M.J., and Burgoyne, P.S. (2001). A Y-encoded subunit of the translation initiation factor Eif2 is essential for mouse spermatogenesis. Nat. Genet. *29*, 49-53.
3. Prosser, H.M., Rzadzinska, A.K., Steel, K.P., and Bradley, A. (2008). Mosaic complementation demonstrates a regulatory role for myosin VIIa in actin dynamics of stereocilia. Mol. Cell. Biol. *28*, 1702-1712.

4. Lane, P.W., and Davisson, M.T. (1990). Patchy fur (*Paf*), a semidominant X-linked gene associated with a high level of X-Y nondisjunction in male mice. J. Hered. *81*, 43-50.
5. Mazeyrat, S., Saut, N., Sargent, C.A., Grimmond, S., Longepied, G., Ehrmann, I.E., Ellis, P.S., Greenfield, A., Affara, N.A., and Mitchell, M.J. (1998). The mouse Y chromosome interval necessary for spermatogonial proliferation is gene dense with syntenic homology to the human AZFa region. Hum Mol Genet *7*, 1713-1724.
6. Gubbay, J., Collignon, J., Koopman, P., Capel, B., Economou, A., Munsterberg, A., Vivian, N., Goodfellow, P., and Lovell-Badge, R. (1990). A gene mapping to the sex-determining region of the mouse Y chromosome is a member of a novel family of embryonically expressed genes. Nature *346*, 245-250.
7. Gubbay, J., Vivian, N., Economou, A., Jackson, D., Goodfellow, P., and Lovell-Badge, R. (1992). Inverted repeat structure of the Sry locus in mice. Proc Natl Acad Sci U S A *89*, 7953-7957.
8. Mahadevaiah, S.K., Odorisio, T., Elliott, D.J., Rattigan, A., Szot, M., Laval, S.H., Washburn, L.L., McCarrey, J.R., Cattanach, B.M., Lovell-Badge, R., et al. (1998). Mouse homologues of the human AZF candidate gene RBM are expressed in spermatogonia and spermatids, and map to a Y chromosome deletion interval associated with a high incidence of sperm abnormalities. Hum Mol Genet *7*, 715-727.
9. Cattanach, B.M., Pollard, C.E., and Hawkes, S.G. (1971). Sex reversed mice : XX and XO males. Cytogenetics *10*, 318-337.
10. Mahadevaiah, S.K., Evans, E.P., and Burgoyne, P.S. (2000). An analysis of meiotic impairment and of sex chromosome associations throughout meiosis in XYY mice. Cytogen. Cell Genet. *89*, 29-37.
11. Russell, L.D., Ettlin, R.A., Hikim, A.P.S., and Clegg, E.D. (1990). Histological and histopathological evaluation of the testis, 1 Edition, (Clearwater, FL : Cache River Press).
12. Ahmed, E.A., and de Rooij, D.G. (2009). Staging of mouse seminiferous tubule cross-sections. Methods Mol. Biol. *558*, 263-277.
13. Barlow, A.L., Benson, F.E., West, S.C., and Hult,n, M.A. (1997). Distribution of the RAD51 recombinase in human and mouse spermatocytes. EMBO J. *16*, 5207-5215.
14. Cocquet, J., Ellis, P.J., Yamauchi, Y., Mahadevaiah, S.K., Affara, N.A., Ward, M.A., and Burgoyne, P.S. (2009). The multicopy gene Sly represses the sex chromosomes in the male mouse germline after meiosis. PLoS Biol *7*, e1000244.
